# Maternal mental health and adverse birth outcomes

**DOI:** 10.1371/journal.pone.0272210

**Published:** 2022-08-31

**Authors:** Falk A. C. Voit, Eero Kajantie, Sakari Lemola, Katri Räikkönen, Dieter Wolke, Daniel D. Schnitzlein

**Affiliations:** 1 Institute of Labour Economics, Leibniz Universität Hannover, Hannover, Germany; 2 Finnish Institute for Health and Welfare (THL), Helsinki, Finland; 3 PEDEGO Research Unit, MRC Oulu, Oulu University Hospital and University of Oulu, Oulu, Finland; 4 Department of Clinical and Molecular Medicine, Norwegian University for Science and Technology, Trondheim, Norway; 5 Fakultät für Psychologie und Sportwissenschaft, Universität Bielefeld, Bielefeld, Germany; 6 Department of Psychology, University of Warwick, Coventry, United Kingdom; 7 Department of Psychology and Logopedics, University of Helsinki, Helsinki, Finland; 8 IZA Institute of Labor Economics, Bonn, Germany; University of Jyvaskyla, FINLAND

## Abstract

Recent research in economics emphasizes the role of in utero conditions for the health endowment at birth and in early childhood and for social as well as economic outcomes in later life. This paper analyzes the relation between maternal mental health during pregnancy and birth outcomes of the child. In particular, we analyze the relationship between maternal mental health during pregnancy and the probability of giving birth preterm (PT), having a newborn at low birth weight (LBW) or being small for gestational age (SGA). Based on large population-representative data from the German Socio-Economic Panel (SOEP) and cohort data from the National Educational Panel Study (NEPS), we present extensive descriptive evidence on the relationship between maternal mental health and preterm birth by carrying out OLS estimates controlling for a wide range of socioeconomic characteristics. In addition, we apply matching estimators and mother fixed effects models, which bring us closer toward a causal interpretation of estimates. In summary, the results uniformly provide evidence that poor maternal mental health is a risk factor for preterm birth and low birth weight in offspring. In contrast, we find no evidence for an relationship between maternal mental health and small for gestational age at birth.

## 1 Introduction

Every year, approximately 15 million children are born preterm (PT) (<37 weeks gestation), and 20 million have low birth weight (LBW) (<2500 g) [1, 2]. Being born PT or with LBW has lasting effects over the life cycle. Previous research has shown that these adverse birth outcomes are associated with lower education, fewer employment chances, health issues, lower socioeconomic status and decreased economic prosperity [3–5]. Psychologists argue that the last trimester of pregnancy is central to brain development, as substantial parts of the cerebral cortex are still developing in that period [6]. Altered brain development in preterm children involves alterations in brain volume, cortical folding and impaired functional networks [7].

The WHO aims to reduce the incidence of low birth weight by 30% by 2025 [8]. As PT birth is highly correlated with LBW, the World Health Organization states: *“An increased awareness of the long-term consequences of preterm birth (at all gestational ages) is required to fashion policies to support these survivors and their families as part of a more generalized improvement in quality of care for those with disabilities in any given country”* [9]. These long-term consequences lead to substantial direct and indirect costs. A study that investigates German data shows that early PT birth is associated with more than 72,000 € of additional health care spending per child in the first year after birth [10] and a systematic review of articles indicates that the association between preterm birth and increased costs is prevalent in various other developed countries [11].

Due to the challenges following preterm birth, increased efforts have been made to identify risks as well as protective and/or resilience factors for prematurity [12]. Identifying and tackling these risk factors is central to suitably prevent, or lower the incidence of preterm birth. Risk factors for having a PT baby include smoking during pregnancy, teenage pregnancy, an interval of fewer than 12 months after a prior birth, birth of twins/multiples, previous PT birth, maternal health and fertility problems [13]. Beyond that, the economic literature has analyzed the impact of diseases such as flu [14], malnutrition [15], pollution [16, 17] and socioeconomic disadvantages [18] as potential risk factors for infants’ birth outcomes. In addition, it is controversly discussed whether economic crisis and unemployment are associated with increased rates of children with adverse birth outcomes [19–21]. Moreover, intense physical exercise and maternal anxiety are further risk factors for having a PT born baby [22].

Maternal mental health during pregnancy might be a further relevant factor to explain adverse birth outcomes. The WHO pointed to poor maternal mental health as a possible risk factor for PT birth and suggested actions to improve the diagnoses and maintenance of maternal mental health during pregnancy by member states [9]. In addition to PT birth and LBW, small for gestational age (SGA) birth is another frequently used indicator of the infant´s health endowment at birth [23, 24]. Accordingly, this article investigates whether poor maternal mental health during pregnancy is associated with a higher risk of LBW, PT and SGA birth.

Previous research in health economics has mainly focused on maternal stress during pregnancy rather than analyzing the mental health of mothers. Since maternal stress during pregnancy is both difficult to measure in a valid way and arguably correlated with various unobserved characteristics, early research was rather descriptive [25, 26]. More recent contributions have used exogenous variation in stress levels to tackle selection problems. Torche (2011) studies the effects of stress on birth outcomes, which was exogenously induced by the 2005 Tarapaca earthquake. She reports that earthquake-induced stress significantly increased the incidence of low birth weight and reduced the average gestational age [27]. Following a comparable approach, Currie and Rossin-Slater (2013) analyze US data on hurricane exposure during pregnancy. Using the geographical variation of hurricanes as a natural experiment for stress, the authors find descriptive evidence for increased abnormal conditions after birth (e.g., being on a ventilator for more than 30 min or meconium aspiration syndrome). However, the effects seem to diminish once causal models are applied [28].

One limitation of these articles is that they lack an objective stress measure and indirectly associate all the variation from these events to higher stress levels or rely on self-reported stress levels. In contrast, Aizer, Stroud and Buka (2016) use cortisol levels as a measure of stress. Applying sibling fixed effects, they show that maternal stress during pregnancy leads to lower-level educational outcomes of the offspring [29]. Persson and Rossin-Slater (2018) study a large US dataset, which includes cortisol levels of mothers during pregnancy. They use the passing of a family member of the mother as an instrumental variable for stress (changes in cortisol levels) and find significant effects on preterm birth and low birth weight in the offspring [30].

Maternal mental health effects on infant health are still under debate. Whereas some authors report adverse effects of poor maternal mental health on birth outcomes [31–33], others do not [34, 35]. In an extensive review of psychological literature, only a quarter of all considered papers identify an adverse impact of maternal mental health problems on infant health at birth [36]. In addition, it remains unknown whether associations between maternal mental health and infant birth outcomes represent a causal relationship or are driven by reversed causality, omitted variables or selection problems.

We contribute to this literature by investigating the effect of maternal mental health during pregnancy on the risk of PT, LBW and SGA birth, controlling for a wide variety of socioeconomic, demographic and health-related characteristics of both the mother and the child. This potential effect of maternal mental health during pregnancy could be seen as one aspect of the prenatal programming of postnatal plasticity [37] and the fetal origins hypothesis [38]. Both hypotheses entail that maternal and family characteristics determine the child´s development, even if it is not yet born [39–41].

Beyond that, we estimate matching and fixed effects models to account for potential endogeneity problems. We also evaluate the internal validity of our results by performing a specification curve analysis and the external validity by analyzing two different datasets from Germany with comparable survey instruments.

Our results show that maternal mental health problems during pregnancy are indeed a substantial risk factor for adverse birth outcomes. This result also holds after the inclusion of a wide range of socioeconomic characteristics as well as physical health measures of the mother and is robust given different identification strategies or data sources.

## 2 Data and descriptive evidence

We use two German datasets to estimate the relevance of mental health problems during pregnancy for the incidence of PT birth: the Socio-Economic Panel (SOEP), a representative household study, and the National Educational Panel Study (NEPS), a cohort study. Both offer highly comparable questions in their mother-newborn surveys but different target populations, giving us the opportunity to test for the external validity of the results obtained from one of the two datasets. For our main analysis, we focus on the SOEP data. The NEPS is used to infer the external validity of the results. The first reason for the SOEP as our data source for the main analysis is that the SOEP includes birth weight and gestational age as continuous variable. Hence, we could calculate SGA based on this information. With the NEPS data this is not possible, because birth weight and gestational age are assessed as categorical variables. Second, only the SOEP includes information on the mental health component score before birth, which is a very important and meaningful variable to balance in the matching procedure. Third and foremost, the SOEP is a panel data set. It contains information from siblings, allowing us to estimate mother fixed effect models. The NEPS as a cohort dataset does not include any information on the birth outcomes of siblings of the individual included in the cohort. The SOEP and the NEPS are two large publicly funded German datasets intended for secondary analysis and widely used for scientific purposes. All participants were individually asked for their consent to participate in the study. In the case of minors, the consent was given by their legal representatives (usually their parents). All statements of consent are stored and archived by the German Institute for Economic Research (DIW) in Berlin for the SOEP data and by the Leibniz Institute for Educational Trajectories in Bamberg for the NEPS data and/or their respective field institutes. The SOEP data is accessible for researchers under permission. Permission is provided by the Scientific Advisory Board of DIW Berlin. In order to prevent harm to the respondents, the data may only be used for specific purposes (scientific research) and factually anonymized. Prior to any disclosure to new users, the institutional data protection officer verifies that the data are only shared for scientific use and are appropriately anonymized and/or protected. We can guarantee that on the participant side there is informed consent and voluntariness. The fact that participation is voluntary is explained to the participants each wave. The NEPS study is conducted under the supervision of the German Federal Commissioner for Data Protection and Freedom of Information (BfDI) and in coordination with the German Standing Conference of the Ministers of Education and Cultural Affairs (KMK) and–in the case of surveys at schools–the Educational Ministries of the respective Federal States. All data collection procedures, instruments and documents were approved by the data protection unit of the Leibniz Institute for Educational Trajectories (LIfBi). The necessary steps are taken to protect participants’ confidentiality according to national and international regulations of data security. Participation in the NEPS study is voluntary and based on the informed consent of participants. This consent to participate in the NEPS study can be revoked at any time.

The SOEP is a representative household panel study that started in 1984 and has involved yearly assessments [42]. Data for newborns and their mothers has been collected from 2000 onwards. The sampling is based on the household, and every household member should be included in the SOEP data. The selection of households in the SOEP aims to be representative of the German population. The SOEP data in version 34 provide birth information of mothers with children born between 2000 and 2017. Our initial sample consists of 4,656 mother-child pairs with information on gestational age and which were observed before and after pregnancy. It also include information on birth weight and information on maternal mental and physical health in the last third of the pregnancy. We analyze three outcome variables.

**Fig 1:**
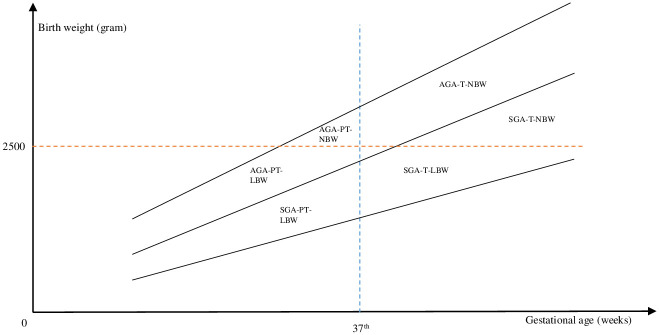
The relationship between preterm birth, LBW and SGA. The figure shows the relationship between the birth outcomes preterm, LBW and SGA. NBW = normal birth weight; LBW = low birth weight; T = term-born; PT = preterm-born; SGA = small for gestational age; AGA = appropriate for gestational age [44].

First, PT is assigned a value of one if a child was born before the completion of the 37^th^ week of gestation and zero otherwise. LBW includes individuals with a birth weight below 2500 g as defined by the WHO. Finally, we estimate the coefficient for mental health during pregnancy on a combined measure of both previous concepts: SGA. SGA assigns a value of one to everyone whose birth weight is below the first decile of a gender- and gestation-specific distribution of birth weight, as suggested by Voigt et al. (2014) [43]. Fig 1 visualizes the three concepts and their overlaps.

Our variable of interest is maternal mental health. In addition, we also include self-reported maternal physical health in our analysis. The exact wording of the survey question is:

*“How were you feeling physically and mentally during the last third of your pregnancy and during the first three months after giving birth?”*

(1 = very good, 2 = good, 3 = bad, 4 = very bad)

All mothers who gave birth in the year before the annual survey interview, which are already part of the study, were asked to provide information on their mental and physical health status. Responding mothers answer separate items for both health variables and both periods after they gave birth. The question has two parts: one asking for mental health status during pregnancy and the other for the mental health after birth. We use the answer on the first item, which is the mental health status during the last third of pregnancy and aggregate the four answer categories to an indicator variable, which takes the value one for a very bad or bad maternal mental health status, and zero otherwise. The same transformation was applied to the physical health variable. We form the measure in this particular way because there is a clear change in meaning between categories two and three (from good to bad). To summarize, the procedure described above yields two indicator variables, one for inferior physical health and one for poor mental health during pregnancy. S1 Fig in S1 File shows the distribution of both health variables, given the sample we use for OLS estimation later. Here, 14% of all mothers report bad or very bad mental health, and 23% report bad or very bad physical health.

In addition to these variables, S1 Table in S1 File summarizes potential risk factors as well as the protective or resilience factors of the mother, which are used as control variables in the regression models. As socioeconomic and demographic characteristics, we include an indicator for higher education (high school diploma or better), average weekly working hours, migration status and marital status of the mother, logarithmic average household income (annually) before birth, household size and an indicator for homeownership. For fertility aspects that are related to previous births, indicators for the child being an older sibling, having a preterm-born sibling, or a twin brother/sister are included. To account for physical health and health behavior, we also include a variable indicating whether mothers ever smoked before birth as well as the physical health indicator discussed previously. Finally, we control for the sex of the newborn. Excluding individuals with missing or implausible values in any of the variables (e.g. birth weight of 7500g) yields a final sample of 1655 mothers and 2141 children, of which 207 children were born PT (9.67%), 149 had a LBW (6.96%), and 263 were SGA births (12.28%). The main reason is that some of the 2141 children are siblings (and some are twins/multiples) and thus share the same mother. Hence, we have less mothers than children in our sample. We decided to keep them in the OLS samples and address this by including information on siblings and twins as a control variables in the OLS models. Official German statistics report comparable rates of preterm birth (8.36%), low birth weight (7.03%) and low birth weight for gestational age (10.00%) [45]. Children in our sample were born between the 24th and 45th weeks of gestation, and their birth weight ranged from 540 grams to 5140 grams. We also included siblings (and twins) in the sample, and we use this information and include it as control variables in our OLS models. The only variables with a considerable amount of missings are the variable “smoked before pregnancy” (2,171) and “working hours of the mother” (1,716).

Maternal mental and physical health differ significantly across the two categories of the LBW and preterm indicator. In the term-born group, 12% of mothers reported a poor mental health status during pregnancy, whereas 30% in the preterm-born group did so. If we consider SGA as an outcome variable, we find no large differences in the share of mothers with a bad or very bad mental health.

S1 Table in S1 File also shows the mean values of our control variables across our outcome categories (PT, LBW, SGA). As expected, we observe that preterm-born individuals show a significant higher rate of low birth weight but no higher risk of small for gestational age. Mothers having a preterm-born child are more likely to have smoked before birth. Moreover, they were slightly less educated in terms of a higher secondary degree and worked significantly more on average before childbirth took place. We observe higher rates of preterm birth given that mothers experienced multiple births or previous preterm births. Mothers having a preterm-born child live in smaller households and are less likely to own property. Preterm-born children live in households with lower logarithmic income. The observed differences in the data replicate the findings of previous research on risk factors for preterm birth. Turning to LBW or SGA, we observe comparable patterns with respect to significant differences of control variables, given a 10%-significance level. The National Educational Panel Study (NEPS) is a multi-cohort study from Germany that mainly focuses on education and related topics. The newborn cohort data (SC-1) contain information on central birth outcomes, comparable to those available in the SOEP, discussed above [46].

The NEPS also contains information on the central control variables or comparable alternatives to those we also use in the SOEP sample described previously. In particular, the question on maternal mental health in the NEPS questionnaire reads:

*“How were you feeling mentally during the last trimester of your pregnancy?”*

(1 = very good, 2 = rather good, 3 = rather bad, 4 = very bad)

After we exclude all cases with missing values in key variables summarized in S2 Table in S1 File, we arrive at a sample of 1,841 children born in 2012. Here, 16% of all mothers reported a bad or very bad mental health status, and 27% reported a bad or very bad physical health status. In the NEPS, which is a cohort design, most births took place in one particular year, whereas the SOEP includes birth information over a period of 18 years. The NEPS focuses on one specific birth cohort. The target population here are individuals born between February 2012 and July 2012, and only household members were surveyed if the baby was between six and eight months old by the time, the interview should take place. The sample we use includes 121 (6.57%) preterm-born children and 107 (5.81%) with a low birth weight. In the NEPS, relative shares are slightly smaller, as reported by official statistics. Since birth weight information was reported as a categorical variable in the NEPS, we could generate the low birth weight indicator but are not able to calculate low birth weight for gestational age.

S2 Table in S1 File presents descriptive statistics for the NEPS sample. In the NEPS, 40% of all mothers with preterm-born children reported poor mental health during pregnancy, and only 15% with a term-born child did so. This difference is also prevalent in the SOEP data, and the same is true for the case of low birth weight. Generally, mothers of children born preterm do not differ significantly from term-born children in this sample, except for the share of mothers who are homeowner. We only observe values reported shortly after birth. This explains the comparable small mean values in the working hour variable because most observed mothers in the NEPS should still be on maternity leave while surveyed. We use the NEPS sample to control for the external validity of the results we obtained in our main analysis with the SOEP data.

## 3 Empirical strategy

In the first step, we estimate OLS models to identify the coefficient of mental health on the three birth outcomes discussed previously. We run OLS regressions in both samples with and without control variables. Formally, the OLS model can be described as:

$${Birth\;outcome}_{i}=b_{o}+b_{1}{MH}_{i}+b_{2}\prime {Controls}_{i}+\varepsilon_{i}$$ (1)

*Birth outcome*_*i*_ consists of one of our three indicators (PT, LBW, SGA). *b*_*o*_ is a constant, and *Controls* is the matrix of control variables, which we discussed previously. *ε*_*i*_ denotes an idiosyncratic independently identically distributed (iid) error term. The parameter of interest is *b*_1_, which yields the association between maternal mental health (*MH*_*i*_) and the respective birth outcome. However, simple OLS estimates might be biased due to omitted variables. Unobservable characteristics could induce a risk of both mental health problems during pregnancy and PT birth. One example would be the unobserved health behavior of the mother, which is related to a higher risk of depressive symptoms or mental health problems in general but also impairs birth outcomes. Nevertheless, OLS estimates are highly informative in terms of the total association between the concepts.

Since our dependent variables are binary, Eq (1) represents a linear probability model (LPM). We decided to base our main analysis on estimated LPMs to ease the interpretation of coefficients. However, the results are virtually identical in a logit model (see S3 and S4 Tables in S1 File).

The SOEP data also enable us to identify biological mothers of the children and estimate our parameter of interest controlling for mother fixed effects. In the mother fixed effects model, we are able to control for all mother-specific unobservable characteristics that do not vary across different births, which brings us slightly closer toward a causal interpretation of estimates. This includes any constant genetic influence the mother has on the birth outcome of the child. A formal description of mother fixed effects models is:

$${Birth\;outcome}_{ij}=c_{o}+c_{1}{MH}_{ij}+c_{2}^{\prime }{Controls}_{ij}+U_{j}+\vartheta_{ij}$$ (2)

Fixed effects models are essentially OLS models with an additional indicator variable *U*_*j*_ for each mother *j* of child *i*. Since we already control for all within-mother variation, we cannot identify coefficients for all variables, which are constant for the mother across births, because their influence is already captured by the fixed effect. *Controls*_*ij*_ is a matrix of control variables for mother *j* and child *i*, which are not constant across births. The coefficient of interest is *c*_1_, which is the association between maternal mental health (*MH*ij) and the respective birth outcome conditional on the included control variables and the mother fixed effect (*U*_*j*_).

One other way to address potential biases is to apply a matching approach. Matching enables us to estimate the Average Treatment Effect on the Treated (ATT) and Average Treatment Effect (ATE) [47]. In our setting, ATT is the causal effect of maternal mental health on the respective birth outcome for mothers with a poor mental health, and ATE is the effect of poor maternal mental health for all mothers.

The intuition behind a matching estimator can be imagined as a two-step approach. In the first step, we predict the probability (propensity score) of a poor mental health status for every mother with a set of socioeconomic and health-related covariates. Second, we compare mothers having an actual premature-born child and those with term-born children such that both mothers have similar probabilities of a mental health problem, which is our treatment. For reasons described later in the text, we apply a kernel-based matching model. Mothers of the treatment group are matched with control group mothers, such that they are comparable in terms of included covariates.

If we compare mothers of the treatment group (*t*) with a weighted average of control group members (*c*) (kernel matching), we estimate the potential counterfactual birth outcome ($\hat{Birth\;outcome}$) given the following formula:

$$\hat{Birth\;outcome}=\frac{\sum_{c\in \left\{MH=0\right\}}W\left(q\right){Birth\;outcome}_{c}}{\sum_{c\in \left\{MH=0\right\}}W\left(q\right)};with\;q=\frac{p_{t}-p_{c}}{h}$$ (3)

Here, *W* denotes the weight of the kernel function we apply, *p* the propensity score and *h* the bandwidth. For our purpose, we use two different kernel functions, Gaussian and Epanechnikov with a fixed bandwidth *h* of 0.06:

$$W_{gau}(q)\propto \text{exp}\left(\frac{-q^{2}}{2}\right)$$ (4)

$$W_{epa}\left(q\right)\propto \left(1-q^{2}\right)if\left|q\right|<1$$ (5)

We decided to stick to the same bandwidth for both kernel matching functions in order to keep the results comparable. Differences between the two kernels should thus not represent differences in the bandwidth selection. The smaller the bandwidth the stronger is the weighting of very similar observations of the treatment and control group, which could influence our results. We also estimated the models with alternative bandwidths (0.04 & 0.08). Results are similar to the results with a bandwidth of 0.06. The matching results indicate that maternal mental health is a risk factor for preterm birth and low birth weight but not for small for gestational age. Given the procedure described above, we calculate the ATT and the ATE for mental health (MH), estimating the average treatment effect on the control group (ATC) as follows [48]:

$$ATT\left(MH\right)=\frac{1}{N_{t}}\sum_{t}\left({Birth\;outcome}_{t}-\frac{\sum_{c}{Birth\;outcome}_{c}W\left(q\right)}{\sum_{c}W\left(q\right)}\right)$$ (6)

$$ATC\left(MH\right)=\frac{1}{N_{c}}\sum_{c}\left(\frac{\sum_{t}{Birth\;outcome}_{t}W\left(q\right)}{\sum_{t}W\left(q\right)}-{Birth\;outcome}_{c}\right)$$ (7)

$$ATE\left(MH\right)=\frac{N_{t}}{N}ATT\left(MH\right)+\frac{N_{c}}{N}ATC\left(MH\right)$$ (8)

If we include all relevant predictors for poor mental health status (conditional independence), we could interpret the ATT and the ATE causally [49]. We computed our results using the software Stata 16 [50] and R [51] as well as packages provided in those software applications.

## 4 Results

Even though OLS estimates may be biased, as argued above, they provide a benchmark for the underlying causal effect. Table 1 presents the results from OLS and mother fixed effects models for all three outcomes we mentioned previously (rows 1–6). The results show a clear pattern: poor maternal mental health in the last trimester of pregnancy significantly raises the risk of having a preterm-born child in all models presented in Table 1. The same is true for LBW but not for SGA. In column (1), we show OLS without any controls included, whereas column (2) shows the OLS coefficients for maternal mental and physical health conditional on all variables discussed in section 2. Even though the inclusion of further control variables helps to explain more of the variance in our outcomes (higher R^2^), it does not change the coefficients for mental or physical health substantially. OLS only produces consistent estimates if the assumptions of the linear probability model hold and are only causally interpretable if the distribution of maternal mental health issues across term and preterm-born children is not affected by any other covariate, which is not included in our model.

**Table 1:** OLS and FE models—SOEP.

	(1)	(2)	(3)	(4)
	OLS^d^	OLS^d^	FE^e^	FE^e^
**Preterm** ^a^				
Poor Mental Health	0.127***	0.095***	0.097***	0.062***
	(0.024)	(0.027)	(0.022)	(0.018)
Poor Physical Health		0.046*		0.014
		(0.020)		(0.016)
**LBW** ^b^				
Poor Mental Health	0.097***	0.078***	0.063***	0.046*
	(0.021)	(0.023)	(0.018)	(0.018)
Poor Physical Health		0.037*		0.022
		(0.016)		(0.016)
**SGA** ^c^				
Poor Mental Health	0.019	0.007	0.035	0.048
	(0.021)	(0.023)	(0.024)	(0.0261)
Poor Physical Health		-0.002		0.001
		(0.019)		(0.023)
Controls		Yes		Yes
Observations	2,141	2,141	2,119	2,119

The table presents OLS and mother fixed effects estimates for the relationship between maternal mental health and our birth outcomes (PT, LBW, SGA) using the SOEP sample. OLS estimates are based on a full information sample of all individuals. Mother fixed effects models are based on the whole sample of mothers with at least two children, without conditioning on full information for all control variables and excluding all pregnancies with multiple births. In the OLS model with controls, we include the mother´s age at birth (and squared), maternal smoking before birth, average working hours of the mother before birth, marital status, education and migration background of the mother, homeownership, household size and income before birth, an indicator for previous preterm births, twin and multiple births, the presence of older siblings, the sex of the child and year and region fixed effects. The mother fixed effects model with controls includes the sex of the child as an indicator for previous preterm birth and one for the presence of an older sibling. Robust standard errors in parentheses.

**** p<0.001,

*** p<0.01,

* p<0.05.

^a^ Preterm birth.

^b^ Low birth weight.

^c^ Small for gestational age.

^d^ Ordinary Least Squares Model

^e^ Mother fixed effects model

If these assumptions hold, poor maternal mental health in the last trimester of pregnancy is related to a 9.5–12.7 percentage point increased risk for PT birth. Accordingly, poor maternal mental health is associated with a 7.8–9.7 percentage point higher risk for LBW. For the SGA outcome, we find no consistent significant maternal mental health coefficients. This might be because many children with low birth weight for gestational age are not born preterm and have no low absolute birth weight. For example, in our sample, 12.25% of term-born and 12.56% of preterm-born children were categorized as SGA. Various children born with low birth weight for gestational age are at the lower end of the gender-birth-week specific birth weight distribution but are not born preterm, have no low birth weight and hence might face no substantial developmental disadvantages at birth. The influence of the additional control variables is as would be expected (see S5 Table in S1 File) With only a few exceptions, we see the same picture with respect to control variables regarding our other two birth outcomes (LBW, SGA).

Columns 3 and 4 present results from the mother fixed effects models, which allow us to control for constant but unobservable maternal characteristics. For the analysis, we drop all mothers who had one child and all twin births since there was no variation in gestational age or the mental health of the mother during pregnancy.

Again, poor maternal mental health increases the risk of preterm birth, but the coefficients are smaller in comparison to those obtained in OLS models, which is what we would expect. This indicates that unobserved factors at the mother level influence both the child´s birth outcomes and the mental health condition of the mother accordingly. The same is true for the case of LBW and partly for SGA. Interestingly, physical health is not significant in the most comprehensive model (column 4) for any of the three outcomes. Here, we also include various birth-related variables that are correlated with maternal physical health. Poor maternal mental health still significantly increased the risk of preterm birth (6.2–9.7 percentage points) and low birth weight (4.6–6.3 percentage points). As is the case in OLS models, we do not find consistent significant coefficients of maternal mental health on the incidence of SGA at the child´s birth. Our fixed effects models show that the relationship between maternal mental health and the child´s birth outcomes (LBW, PT) is not entirely explained by mother-specific characteristics such as genetics (see also S6 Table in S1 File for the full table of results).

In the absence of a suitable instrumental variable for the mental health status of the mother during pregnancy, we also apply matching estimators to get closer toward causality. We use kernel-based matching algorithms, Gaussian and Epanechnikov, and match on all covariates we used as control variables in our OLS models. Moreover, we also use the mental health component summary score (mcs) before birth as a covariate to balance our treatment and control groups. This score is calculated using multiple subscales representing different dimensions of mental health. The SOEP includes four subscales, which cover general mental health, emotional role, social functioning and vitality. Higher mcs scores represent a better mental health condition. We compare birth outcomes for mothers with comparable mcs scores before birth. For our estimation, we use the same sample as in the OLS analysis, excluding observations with missing information on the mcs scores before birth.

The results are summarized in Table 2 (see also S7 Table in S1 File for the full table of results). The estimates for the ATE and the ATT confirm our previous results. Mental health is a risk factor for PT and LBW but not for SGA. We estimate the relation based on kernel matching since we have better common support compared to nearest-neighbor matching. Predicting mental health via a Gaussian kernel always leads to perfect common support by design (see [52] for more details on different matching techniques). S8 Table in S1 File presents the pre- and after-matching differences for the variables we use in our estimation of mothers with poor mental health during pregnancy and those without. It is evident that both matching procedures drastically reduce the differences in almost all the considered variables.

**Table 2:** Matching estimates for treatment effects–SOEP.

	(1) GAU^f^	(2) EPA^g^
**Preterm** ^a^		
ATE^d^	0.113*	0.144***
	(0.057)	(0.034)
ATT^e^	0.086**	0.093**
	(0.034)	(0.029)
**LBW** ^b^		
ATE^d^	0.130**	0.091**
	(0.055)	(0.036)
ATT^e^	0.072**	0.065**
	(0.030)	(0.027)
**SGA** ^c^		
ATE^d^	0.031	-0.009
	(0.040)	(0.027)
ATT^e^	0.003	-0.017
	(0.025)	(0.027)
N	2,134	2,134
support	1	0.838

The table presents matching estimates for the Average Treatment Effect (ATE) and Average Treatment Effect on the Treated (ATT) of maternal mental health on our birth outcomes (PT, LBW, SGA) using the SOEP sample. We matched all covariates presented in S8 Table in S1 File as well as survey years, federal states and a squared term of maternal age (not indicated in S8 Table in S1 File). Bootstrapped standard errors in parentheses (100 replications).

*** p<0.001,

** p<0.01,

* p<0.05.

The sample size is different because the mcs scores are added as a covariate to match individuals.

^a^ Preterm birth.

^b^ Low birth weight.

^c^ Small for gestational age.

^d^ Average Treatment Effect.

^e^ Average Treatment Effect on the Treated.

^f^ Gaussian Kernel.

^g^ Epanechnikov Kernel.

In the sample, we analyze 21% of the mothers with poor mental health during pregnancy who had a preterm-born child, whereas only 8% of those without mental health problems had a child born preterm. The estimated ATE of a poor maternal mental health status during pregnancy on the risk of preterm birth equals 11.3 percentage points given Gaussian kernel matching. The ATT indicates that for mothers with poor health, the risk of preterm birth is 8.6 percentage points higher. The second panel shows the ATE and ATT for an Epanechnikov kernel matching approach. Again, we find a significant ATE indicating that poor maternal mental health increases the risk of preterm birth by 14.4 percentage points. The ATT remains significant using an Epanechnikov kernel, as it is the case for the Gaussian kernel and indicates 9.3 percentage points higher risk for preterm birth. If we now turn to the case of LBW, we see a comparable picture. The ATT and ATT of poor mental health status on LBW ranges between 6.5 and 13.0 percentage points. Again, SGA is not related to the mental health status of the mother in any of the models.

The differences in the results using the two kernels could be partly explained by their mechanics. Whereas the Gaussian kernel includes all observations in the estimation and weighs them according to their differences in their estimated probability of having a poor mental health status, the Epanechnikov kernel excludes some that do not meet the support criteria. Excluding these distinct observations reduces the uncertainty in our estimates for the ATC, since 16% of the control group but only 2% of the treatment group are affected. Because the control group only affects estimates for the ATE (via the ATC) the standard errors of the ATE are drastically reduced, but the ATT errors remain relatively stable.

The results are consistent with those presented previously. We find significant ATTs and ATEs for the outcomes preterm and LBW but not for SGA. In summary, propensity score matching suggests that there is a causal component within the estimates we obtained applying OLS.

## 5 Robustness

An important feature of our study is the estimation of the association between maternal mental health and birth outcomes in two independently surveyed observational datasets. Both studies provide highly comparable measures of birth outcomes and control variables, which gives us the opportunity to compare estimates across two different samples from Germany.

S9 Table in S1 File contains OLS estimates for mental and physical health for the NEPS sample. The results show the same clear pattern as in the SOEP sample. Mental and physical health are both significant risk factors for preterm birth. As in the SOEP, the inclusion of control variables does not drastically alter either the coefficient size or significance. Reporting poor mental health is associated with a 9.3–11.2 percentage point higher risk for preterm birth, which is in the range of the OLS coefficients based on the SOEP sample presented above. Estimates for LBW are also very similar in the NEPS and the SOEP data. The poor mental health status of the mother is associated with a 6.5–7.3 percentage point higher risk of having a newborn with a low birth weight. In summary, OLS estimates in both samples are very comparable, which indicates that they are externally valid for the case of Germany. As discussed above, we cannot calculate the SGA because birth weight is a categorical variable in the NEPS, and we would need a continuous measure here.

In addition to selection issues and unobserved characteristics, we want to address another potential threat, which is reverse causality. In both datasets, mothers reported their mental health status in the last trimester of pregnancy in an interview after birth. Therefore, it might be the case that mothers reported a poor mental health status because they had a child with comparably low birth weight or one born preterm. To address this problem, we run an interaction model with another mental health measure, the mental health component summary score (mcs), before pregnancy and after birth using the SOEP data. Dimensions forming the mcs-score are surveyed every second year in the SOEP [42], so we could infer how mental health changes from the years before birth to those afterward with a difference-in-differences (DiD) framework.

We exclude births for which we have only one observation either from the period before pregnancy or after birth. Overall, our sample includes 605 mothers and 770 births (*i*), which are potentially surveyed every second year between 2002 and 2016 (*t*). For each birth, we analyzed at least two observations of the maternal mcs, one before pregnancy and one after birth. A single mother could be included multiple times, if her mcs score was surveyed multiple times before and after birth and/or she had more than one child. The final sample consists of 3,123 mother-birth-year combinations for which we estimate the following equation:

$${mcs}_{it}=d_{0}+d_{1}*{Preterm}_{i}+d_{2}*{Birth}_{i}+d_{3}*{Birth}_{i}*{Preterm}_{i}+d_{4}^{\prime }{Controls}_{it}+\omega_{it}$$ (9)

*d*_1_ captures the average difference in the mcs between mothers having preterm and term-born babies, *d*_2_ the difference between mothers’ mcs before and after pregnancy. The vector *d*_4_ represents the influence of control variables included in the model. The coefficient of interest is *d*_3_. It shows whether there is a systematic difference in the change of mcs scores between mothers of preterm- and term-born children. It helps to quantify the magnitude of the reverse causality problem in our estimates. We would expect a positive (negative) coefficient if mothers of preterm-born children have systematically higher (lower) mcs scores. If this is the case, mothers of preterm-born children would have a different mental health condition after birth, and our estimates for maternal mental health in the last trimester of pregnancy would represent this fact.

S10 Table in S1 File summarizes coefficient estimates for *d*_1_, *d*_2_ and *d*_3_ and the estimates for a set of controls, which we also use in the OLS models. The analysis is based on the full sample of mothers, in which we have information on the mcs, all control variables included in the final model and on all birth outcomes we consider. We find no evidence of differences in changes of mothers’ mental health from the period before pregnancy to the time after birth between mothers of preterm and term-born children. The coefficients are negative but insignificantly different from zero. Therefore, our results suggest that preterm birth is not associated with worsened mental health score after birth. We do not find evidence for a reversed causality pattern in our data. Consequently, our estimates for maternal mental health status in the last trimester of birth seem not to be driven by reverse causality, since the mental health of mothers with preterm-born children is not altered systematically after birth. In addition, the mcs score and our measure of subjective mental health during pregnancy are strongly related to each other. Before birth, the last observed mcs socore was 3.1 points (*p* < 0.01) lower if mothers reported a bad or very mental health during pregnancy than the mcs score of those who did not.

Furthermore, we want to address the selectivity of our results to the selection of control variables, the identification strategy and to the selection of different subsamples based on SOEP data. To do so, we perform a specification curve analysis [53, 54] varying control parameter combinations, using different outcomes, identification strategies and estimating the respective models in different subsamples. Afterward, we graph the estimates and confidence intervals, which enables us to infer on the robustness of our results with respect to the specification we choose.

Traditionally, it is to some degree the researcher’s freedom to decide which specification to report within the article. The purpose of a specification curve analysis is to reduce the research degree of freedom and present the results of various specifications in an aggregated curve. Given the curve, it is straightforward to evaluate whether the reported results are similar in other specifications, which are not directly shown in the article. Proceeding in this manner, we avoid reporting only selected models, which generate significant estimates for our variable of interest.

First, we estimate the relationship between maternal mental health and our three birth outcomes. Moreover, we use the SGA calculated on the basis of international standards and an indicator using height at birth instead of the birth weight to calculate the SGA [43, 55, 56]. Again, we use both the German and international standards, while we consider the height at birth. We also vary five sets of control variables, five different samples, and identify the coefficients for maternal mental health estimating fixed effects, matching and OLS models. All aspects that could potentially vary (sample, identification, control variables, outcomes) are listed in S11 Table in S1 File.

S2 Fig in S1 File graphs the specification curve. At the vertical axis of the figure is a legend indicating which outcomes, estimation technique, sample and control combinations the respective estimate represents. In total, the specification curve visualizes 1,110 estimates. Nearly all estimates that are insignificantly different from zero (485 of 1,110) are those that are related to the SGA as an outcome variable (477 of 740). For preterm birth, only four coefficients are not significant given a 5% threshold, whereas 181 coefficients are significant. LBW shows a comparable pattern, and nearly all estimates are significant and positive.

The specification curve analysis supports our main finding of a negative association between poor maternal mental health and the birth outcomes of the offspring if we consider LBW or preterm birth. For SGA, we find no consistent evidence that maternal mental health is a risk factor.

## 6 Conclusion

Our main finding is that poor maternal mental health in the last trimester of pregnancy is associated with inferior birth outcomes (PT and LBW). The result is robust across many specifications and is prevalent in two different datasets from Germany. However, we find no consistent association once we consider SGA as an outcome. This leads us to the conclusion that associations between low birth weight and maternal mental health seem to work through associations between maternal mental health and gestational age. However, children affected by a poor maternal mental health status of the mother do not seem to be intrauterine growth retarded with respect to weight gains during pregnancy. Our results are consistent with the fetal origins hypothesis. Children exposed to poor maternal mental health in the womb have a higher risk of showing adverse birth outcomes. Their mothers are at higher risk of having a preterm-born child or a newborn with low birth weight.

Our results suggest that maternal mental health should be prominently mentioned as a risk factor for preterm birth. Medical professionals should include the diagnostics of mental health problems of pregnant women as part of the recommended standard prenatal care examination. Our results emphasize the importance of the WHO recommendations, which already stated in 2012 that the improvement of mental health problems during pregnancy should be targeted to enhance birth outcomes of the newborn. It is important to mention that additional capacities to improve mental health care during pregnancy must be financed by additional public spending, at least in countries with a public health care system.

However, current policy in Germany seems to target the difficulties of parents after they have a preterm-born child rather than the prevention of the incidence of preterm birth itself. For example, in 2020, the German parliament introduced a new law, which increases the maximum time parental allowance is granted by a month if the child was born six weeks before the expected date of birth. While this is welcome support of families, given the substantial indirect and direct costs of adverse birth outcomes, this should be accompanied by efforts to prevent preterm births. Improving the mental health status of mothers during pregnancy could be an essential factor for this prevention. A better supply of midwife services throughout pregnancy with a special focus on the detection and maintenance of maternal mental health problems could be one part of such a prevention strategy. Moreover, one could think of prioritizing pregnant women for psychological services.

Our study has a number of limitations. First, we are not able to use any quasi-experimental variation in mental health to estimate causal effects for birth outcomes. Nevertheless, by applying fixed effects and propensity score matching models, we arguably remove some of the selection and endogeneity problems a purely descriptive analysis would suffer from. Second, our study relies on observational samples from Germany. Even though estimates are similar in two independent data sets, it would be interesting to replicate our result with large administrative data. Analyzing administrative data is promising, since the SOEP and the NEPS are two of the largest German datasets with birth outcomes and mental health measures included, and still the sample sizes are comparably small. One could think of linking medical records on mental health treatments in pregnancy to administrative data on birth outcomes to replicate our results. This leaves room for further research. Nevertheless, not all pregnant women with mental health problems will benefit from psychological care services or are willing to do so. Therefore, studying observational data will remain quite important in the context of mental health in the future. Analyzing both medical health treatment (e.g., therapy or psychopharmaca medication) and self-reported mental health status during pregnancy in one study would be of great interest to see how effective interventions are able to reduce the risk of adverse birth outcomes induced by mental health problems. Third, we could not control for all confounding variables, which influence the relationship between maternal mental health and adverse birth outcomes. Even though we removed all time constant maternal confounders in the fixed effects models, we cannot capture all time variant variables which are important. In addition to that, it is important to emphasize that we did not control for any paternal confounders. We fully abstract from the influence of fathers.

To further infer the external validity of our results, it would be interesting to perform an international comparison. This could answer the question whether our results could be generalized or whether they are driven by country-specific characteristics such as differences in public health care systems or cultural aspects. To perform an international analysis, it would be beneficial to have an international dataset with comparable measures of the most important variables, especially mental health. Nevertheless, it would be fruitful to consider the harmonization of national datasets and analyze the role of mental health during pregnancy for birth outcomes in an international context. One could consider analyzing multiple national datasets with a so-called individual participant data (IPD) meta-analysis, which is frequently used in social science. Despite the lack of an international context, our results for Germany indicate that mental health problems during pregnancy are a key risk factor for inferior birth outcomes.

## Supporting information

S1 File Appendix including all supporting information mentioned in the text. The file includes S1 and S2 Figs as well as the S1 to S11 Tables which are mentioned in the text.
(DOCX)
