# Supplementary material for: Maternal mental health and adverse birth outcomes
Source: PLoS One. 2022 Aug 31;17(8):e0272210. doi: 10.1371/journal.pone.0272210 (PMC9432739; doi:10.1371/journal.pone.0272210)
Supplement: S1 File — The file includes S1 and S2 Figs as well as the S1 to S11 Tables which are mentioned in the text. (DOCX) [file pone.0272210.s001.docx]

**Online Appendix:**

*Figure S1: Distribution of maternal mental and physical health – SOEP*

*Table S1: T-test for zero mean difference (SOEP)*

| **Variables** | **No PT^a^** | **PT^a^** | **p** | **No LBW^b^** | **LBW^b^** | **P** | **AGA^d^** | **SGA^c^** | **p** |
| --- | --- | --- | --- | --- | --- | --- | --- | --- | --- |
| PT^a^ | . | . | . | 0.054 | 0.664 | <0.001 | 0.096 | 0.099 | 0.900 |
|  | . | . |  | (0.227) | (0.474) |  | (0.295) | (0.299) |  |
| LBW^b^ | 0.026 | 0.478 | <0.001 | . | . | . | 0.039 | 0.285 | <0.001 |
|  | (0.159) | (0.501) |  | . | . |  | (0.195) | (0.452) |  |
| SGA^c^ | 0.123 | 0.126 | 0.900 | 0.094 | 0.503 | <0.001 | . | . | . |
|  | (0.328) | (0.332) |  | (0.292) | (0.502) |  | . | . |  |
| Poor Mental Health | 0.124 | 0.300 | <0.001 | 0.128 | 0.309 | <0.001 | 0.138 | 0.160 | 0.365 |
|  | (0.329) | (0.459) |  | (0.334) | (0.464) |  | (0.345) | (0.367) |  |
| Poor Physical Health | 0.219 | 0.411 | <0.001 | 0.214 | 0.436 | <0.001 | 0.225 | 0.259 | 0.247 |
|  | (0.407) | (0.493) |  | (0.410) | (0.498) |  | (0.418) | (0.439) |  |
| Age mother | 31.342 | 31.329 | 0.971 | 31.308 | 31.772 | 0.268 | 31.33 | 31.418 | 0.799 |
|  | (5.047) | (5.060) |  | (5.057) | (4.903) |  | (5.011) | (5.305) |  |
| Smoked before pregnancy | 0.221 | 0.300 | 0.020 | 0.227 | 0.255 | 0.449 | 0.221 | 0.285 | 0.030 |
|  | (0.415) | (0.459) |  | (0.419) | (0.437) |  | (0.415) | (0.452) |  |
| Marital Status | 0.625 | 0.647 | 0.517 | 0.623 | 0.671 | 0.237 | 0.635 | 0.570 | 0.049 |
|  | (0.484) | (0.479) |  | (0.485) | (0.471) |  | (0.482) | (0.496) |  |
| Higher education | 0.500 | 0.435 | 0.074 | 0.493 | 0.503 | 0.808 | 0.497 | 0.468 | 0.368 |
|  | (0.500) | (0.497) |  | (0.500) | (0.502) |  | (0.500) | (0.500) |  |
| Working hours (mother) | 34.063 | 36.093 | 0.005 | 34.032 | 37.289 | <0.001 | 34.048 | 35.77 | 0.011 |
|  | (10.666) | (9.676) |  | (10.728) | (7.950) |  | (10.639) | (10.115) |  |
| Sex | 0.496 | 0.556 | 0.106 | 0.502 | 0.503 | 0.975 | 0.507 | 0.468 | 0.234 |
|  | (0.500) | (0.498) |  | (0.500) | (0.502) |  | (0.500) | (0.500) |  |
| Older sibling | 0.559 | 0.512 | 0.197 | 0.561 | 0.470 | 0.033 | 0.569 | 0.456 | 0.001 |
|  | (0.497) | (0.501) |  | (0.496) | (0.501) |  | (0.495) | (0.499) |  |
| Previous PT^a^ | 0.036 | 0.068 | 0.082 | 0.039 | 0.047 | 0.643 | 0.038 | 0.049 | 0.410 |
|  | (0.187) | (0.252) |  | (0.193) | (0.212) |  | (0.191) | (0.217) |  |
| Twin (multiple birth) | 0.020 | 0.135 | <0.001 | 0.016 | 0.242 | <0.001 | 0.019 | 0.118 | <0.001 |
|  | (0.141) | (0.343) |  | (0.124) | (0.430) |  | (0.137) | (0.323) |  |
| Migration background | 0.237 | 0.271 | 0.299 | 0.244 | 0.181 | 0.058 | 0.244 | 0.209 | 0.192 |
|  | (0.425) | (0.445) |  | (0.430) | (0.386) |  | (0.430) | (0.407) |  |
| Homeowner | 0.351 | 0.295 | 0.094 | 0.345 | 0.349 | 0.929 | 0.347 | 0.335 | 0.687 |
|  | (0.477) | (0.457) |  | (0.476) | (0.478) |  | (0.476) | (0.473) |  |
| Household size | 2.814 | 2.662 | 0.054 | 2.825 | 2.450 | <0.001 | 2.824 | 2.624 | 0.002 |
|  | (1.041) | (1.076) |  | (1.054) | (0.834) |  | (1.053) | (0.968) |  |
| Household income (log) | 10.441 | 10.369 | 0.017 | 10.434 | 10.438 | 0.907 | 10.439 | 10.394 | 0.087 |
|  | (0.407) | (0.406) |  | (0.407) | (0.412) |  | (0.408) | (0.399) |  |

The table shows the p-values given t-tests for different means of important variables across the three birth outcomes (PT, LBW, SGA) using SOEP v34. Standard errors in parenthesis. N = 2141.

^a^ Preterm birth.

^b^ Low birth weight.

^c^ Small for gestational age.

^d^ Appropriate for gestational age.

*Table S2: T-test for zero mean difference (NEPS)*

| **Variables** | **No PT^a^** | **PT^a^** | **P** | **No LBW^b^** | **LBW^b^** | **P** |
| --- | --- | --- | --- | --- | --- | --- |
| PT^a^ | . | . | . | 0.033 | 0.598 | <0.001 |
|  | . | . |  | (0.178) | (0.493) |  |
| LBW^b^ | 0.025 | 0.529 | <0.001 | . | . | . |
|  | (0.156) | (0.501) |  | . | . |  |
| Poor Mental Health | 0.147 | 0.397 | <0.001 | 0.153 | 0.336 | <0.001 |
|  | (0.354) | (0.491) |  | (0.360) | (0.475) |  |
| Poor Physical health | 0.252 | 0.479 | <0.001 | 0.257 | 0.430 | 0.001 |
|  | (0.434) | (0.502) |  | (0.437) | (0.497) |  |
| Migration background | 0.369 | 0.397 | 0.552 | 0.373 | 0.346 | 0.575 |
|  | (0.483) | (0.491) |  | (0.484) | (0.478) |  |
| Working hours (mother) | 2.689 | 2.545 | 0.864 | 2.698 | 2.374 | 0.687 |
|  | (8.667) | (8.886) |  | (8.720) | (8.012) |  |
| Sex | 0.495 | 0.545 | 0.289 | 0.500 | 0.477 | 0.641 |
|  | (0.500) | (0.500) |  | (0.500) | (0.502) |  |
| Household income (log) | 10.554 | 10.525 | 0.486 | 10.549 | 10.606 | 0.219 |
|  | (0.443) | (0.448) |  | (0.442) | (0.462) |  |
| Age mother | 40.396 | 39.496 | 0.105 | 40.365 | 39.879 | 0.419 |
|  | (5.942) | (5.862) |  | (5.935) | (6.031) |  |
| Household size | 3.576 | 3.570) | 0.942 | 3.574 | 3.598 | 0.735 |
|  | (0.820) | (0.773) |  | (0.823) | (0.712) |  |
| Higher education | 0.595 | 0.570 | 0.601 | 0.589 | 0.654 | 0.177 |
|  | (0.491) | (0.497) |  | (0.492) | (0.478) |  |
| Homeowner | 0.517 | 0.430 | 0.065 | 0.515 | 0.449 | 0.185 |
|  | (0.500) | (0.497) |  | (0.500) | (0.500) |  |

The table shows the p-values given t-tests for different means of important variables across the two birth outcomes (PT, LBW) using NEPS SC-1. Standard errors in parenthesis. N = 1,841. We cannot calculate the SGA in the NEPs, since birth weight is a categorical variable, and we would need a continuous measure to do so.

^a^ Preterm birth.

^b^ Low birth weight.

*Table S3: Marginal effects at sample means given logit estimation*

| **VARIABLES** | **(1) PT^a^** | **(2) PT^a^** | **(3) LBW^b^** | **(4) LBW^b^** | **(5) SGA^c^** | **(6) SGA^c^** |
| --- | --- | --- | --- | --- | --- | --- |
| **Poor Mental Health** | 0.127*** | 0.077** | 0.097*** | 0.058** | 0.019 | 0.006 |
| SE | 0.024 | 0.025 | 0.021 | 0.021 | 0.021 | 0.020 |
| T | 5.267 | 3.048 | 4.520 | 2.777 | 0.909 | 0.278 |
| P | <0.001 | 0.002 | <0.001 | 0.005 | 0.363 | 0.781 |
| CI | 0.080 - 0.175 | 0.027 - 0.126 | 0.055 - 0.139 | 0.017 - 0.099 | -0.022 - 0.061 | -0.034 - 0.046 |
| **Poor Physical Health** | . | 0.043* | . | 0.032* | . | -0.000 |
| SE | . | 0.018 | . | 0.013 | . | 0.016 |
| T | . | 2.427 | . | 2.388 | . | -0.018 |
| P | . | 0.015 | . | 0.017 | . | 0.986 |
| CI | . | 0.008 - 0.077 | . | 0.006 - 0.058 | . | -0.033 - 0.032 |
| **Age Mother** | . | 0.002 | . | 0.002 | . | 0.002 |
| SE | . | 0.001 | . | 0.001 | . | 0.001 |
| T | . | 1.751 | . | 1.649 | . | 1.737 |
| P | . | 0.080 | . | 0.099 | . | 0.082 |
| CI | . | 0.000 - 0.005 | . | 0.000 - 0.003 | . | 0.000 - 0.005 |
| **Sex** | . | 0.019 | . | 0.001 | . | -0.015 |
| SE | . | 0.012 | . | 0.008 | . | 0.013 |
| T | . | 1.681 | . | 0.124 | . | -1.205 |
| P | . | 0.093 | . | 0.901 | . | 0.228 |
| CI | . | -0.003 - 0.042 | . | -0.015 - 0.017 | . | -0.041 - 0.010 |
| **Twin (multiple birth)** | . | 0.264*** | . | 0.382*** | . | 0.351*** |
| SE | . | 0.068 | . | 0.077 | . | 0.067 |
| T | . | 3.874 | . | 4.985 | . | 5.272 |
| P | . | <0.001 | . | <0.001 | . | <0.001 |
| CI | . | 0.131 - 0.398 | . | 0.232 - 0.533 | . | 0.221 - 0.482 |
| **Previous PT^a^** | . | 0.080 | . | 0.044 | . | 0.079 |
| SE | . | 0.045 | . | 0.032 | . | 0.046 |
| T | . | 1.788 | . | 1.375 | . | 1.702 |
| P | . | 0.074 | . | 0.169 | . | 0.089 |
| CI | . | -0.008 - 0.167 | . | -0.019 - 0.107 | . | -0.012 - 0.170 |
| **Older Sibling** | . | -0.030 | . | -0.019 | . | -0.058** |
| SE | . | 0.017 | . | 0.012 | . | 0.018 |
| T | . | -1.740 | . | -1.568 | . | -3.238 |
| P | . | 0.082 | . | 0.117 | . | 0.001 |
| CI | . | -0.064 - 0.004 | . | -0.042 - 0.005 | . | -0.093 - -0.023 |
| **Smoked before pregnancy** | . | 0.016 | . | 0.004 | . | 0.019 |
| SE | . | 0.015 | . | 0.010 | . | 0.018 |
| T | . | 1.062 | . | 0.408 | . | 1.093 |
| P | . | 0.288 | . | 0.683 | . | 0.274 |
| CI | . | -0.013 - 0.045 | . | -0.015 - 0.023 | . | -0.015 - 0.054 |
| **Migration background** | . | 0.008 | . | -0.016 | . | -0.021 |
| SE | . | 0.014 | . | 0.009 | . | 0.014 |
| T | . | 0.588 | . | -1.684 | . | -1.488 |
| P | . | 0.556 | . | 0.092 | . | 0.137 |
| CI | . | -0.019 - 0.035 | . | -0.034 - 0.003 | . | -0.049 - 0.007 |
| **Marital Status** | . | 0.016 | . | 0.014 | . | -0.012 |
| SE | . | 0.012 | . | 0.008 | . | 0.015 |
| T | . | 1.299 | . | 1.692 | . | -0.775 |
| P | . | 0.194 | . | 0.091 | . | 0.438 |
| CI | . | -0.008 - 0.040 | . | -0.002 - 0.031 | . | -0.041 - 0.018 |
| **Higher Education** | . | -0.009 | . | -0.002 | . | -0.004 |
| SE | . | 0.013 | . | 0.009 | . | 0.015 |
| T | . | -0.729 | . | -0.257 | . | -0.283 |
| P | . | 0.466 | . | 0.797 | . | 0.777 |
| CI | . | -0.034 - 0.015 | . | -0.020 - 0.015 | . | -0.034 - 0.025 |
| **Working hours mother** | . | 0.001* | . | 0.001* | . | 0.001 |
| SE | . | 0.001 | . | 0.000 | . | 0.001 |
| T | . | 2.270 | . | 2.255 | . | 1.227 |
| P | . | 0.023 | . | 0.024 | . | 0.220 |
| CI | . | 0.000 - 0.003 | . | 0.000 - 0.002 | . | -0.001 - 0.002 |
| **Household income (log)** | . | -0.031 | . | -0.007 | . | -0.034 |
| SE | . | 0.016 | . | 0.012 | . | 0.020 |
| T | . | -1.915 | . | -0.545 | . | -1.739 |
| P | . | 0.056 | . | 0.586 | . | 0.082 |
| CI | . | -0.063 - 0.001 | . | -0.031 - 0.017 | . | -0.073 - 0.004 |
| **Homeowner** | . | -0.013 | . | 0.004 | . | 0.011 |
| SE | . | 0.013 | . | 0.010 | . | 0.016 |
| T | . | -1.070 | . | 0.455 | . | 0.721 |
| P | . | 0.285 | . | 0.649 | . | 0.471 |
| CI | . | -0.038 - 0.011 | . | -0.014 - 0.023 | . | -0.019 - 0.042 |
| **Household size** | . | -0.004 | . | -0.015* | . | -0.001 |
| SE | . | 0.008 | . | 0.006 | . | 0.007 |
| T | . | -0.500 | . | -2.541 | . | -0.081 |
| P | . | 0.617 | . | 0.011 | . | 0.936 |
| CI | . | -0.020 - 0.012 | . | -0.026 - -0.003 | . | -0.015 - 0.013 |
| **Year & Federal State** |  | Yes |  | Yes |  | Yes |
| **R^2^** | 0.029 | 0.111 | 0.028 | 0.200 | <0.001 | 0.068 |
| **N** | 2,141 | 2,128 | 2,141 | 2,141 | 2,141 | 2,141 |
| Standard errors in parentheses. 13 individuals excluded, since one regional fixed effect predicts preterm perfectly. *** p<0.001, ** p<0.01, * p<0.05. Controls are the same variables as in the OLS model (Table 1).  ^a^ Preterm birth.  ^b^ Low birth weight.  ^c^ Small for gestational age. | | | | | | |

*Table S4: Average marginal effects given logit estimation*

| **VARIABLES** | **(1) PT^a^** | **(2) PT^a^** | **(3) LBW^b^** | **(4) LBW^b^** | **(5) SGA^c^** | **(6) SGA^c^** |
| --- | --- | --- | --- | --- | --- | --- |
| **Poor Mental Health** | 0.127*** | 0.080** | 0.097*** | 0.067** | 0.019 | 0.006 |
| SE | 0.024 | 0.025 | 0.021 | 0.022 | 0.021 | 0.022 |
| T | 5.267 | 3.255 | 4.520 | 3.114 | 0.909 | 0.278 |
| P | <0.001 | 0.001 | <0.001 | 0.002 | 0.363 | 0.781 |
| CI | 0.080 - 0.175 | 0.032 - 0.129 | 0.055 - 0.139 | 0.025 - 0.109 | -0.022 - 0.061 | -0.037 - 0.050 |
| **Poor Physical Health** | . | 0.046* | . | 0.039* | . | -0.000 |
| SE | . | 0.019 | . | 0.015 | . | 0.018 |
| T | . | 2.479 | . | 2.517 | . | -0.018 |
| P | . | 0.013 | . | 0.012 | . | 0.986 |
| CI | . | 0.010 - 0.082 | . | 0.009 - 0.069 | . | -0.036 - 0.035 |
| **Age Mother** | . | 0.002 | . | 0.002 | . | 0.003 |
| SE | . | 0.001 | . | 0.001 | . | 0.002 |
| T | . | 1.803 | . | 1.674 | . | 1.663 |
| P | . | 0.071 | . | 0.094 | . | 0.096 |
| CI | . | 0.000 - 0.005 | . | 0.000 - 0.004 | . | 0.000 - 0.006 |
| **Sex** | . | 0.022 | . | 0.001 | . | -0.017 |
| SE | . | 0.013 | . | 0.011 | . | 0.014 |
| T | . | 1.715 | . | 0.124 | . | -1.208 |
| P | . | 0.086 | . | 0.901 | . | 0.227 |
| CI | . | -0.003 - 0.046 | . | -0.020 - 0.022 | . | -0.045 - 0.011 |
| **Twin (multiple birth)** | . | 0.259*** | . | 0.370*** | . | 0.353*** |
| SE | . | 0.061 | . | 0.063 | . | 0.063 |
| T | . | 4.247 | . | 5.836 | . | 5.621 |
| P | . | <0.001 | . | <0.001 | . | <0.001 |
| CI | . | 0.139 - 0.379 | . | 0.246 - 0.494 | . | 0.230 - 0.475 |
| **Previous PT** | . | 0.083 | . | 0.053 | . | 0.048 |
| SE | . | 0.044 | . | 0.036 | . | 1.755 |
| T | . | 1.889 | . | 1.457 | . | 0.079 |
| P | . | 0.059 | . | 0.145 | . | 0.048 |
| CI | . | -0.003 - 0.170 | . | -0.018 - 0.124 | . | -0.010 - 0.177 |
| **Older Sibling** | . | -0.033 | . | -0.023 | . | -0.063*** |
| SE | . | 0.019 | . | 0.014 | . | 0.019 |
| T | . | -1.760 | . | -1.631 | . | -3.341 |
| P | . | 0.078 | . | 0.103 | . | 0.001 |
| CI | . | -0.070 - 0.004 | . | -0.052 - 0.005 | . | -0.099 - -0.026 |
| **Smoked before pregnancy** | . | 0.018 | . | 0.005 | . | 0.021 |
| SE | . | 0.016 | . | 0.013 | . | 0.019 |
| T | . | 1.073 | . | 0.412 | . | 1.103 |
| P | . | 0.283 | . | 0.680 | . | 0.270 |
| CI | . | -0.015 - 0.050 | . | -0.019 - 0.030 | . | -0.016 - 0.059 |
| **Migration background** | . | 0.009 | . | -0.021 | . | -0.023 |
| SE | . | 0.015 | . | 0.012 | . | 0.016 |
| T | . | 0.592 | . | -1.668 | . | -1.484 |
| P | . | 0.554 | . | 0.095 | . | 0.138 |
| CI | . | -0.021 - 0.039 | . | -0.045 - 0.004 | . | -0.054 - 0.007 |
| **Marital Status** | . | 0.018 | . | 0.019 | . | -0.013 |
| SE | . | 0.014 | . | 0.011 | . | 0.016 |
| T | . | 1.279 | . | 1.651 | . | -0.781 |
| P | . | 0.201 | . | 0.099 | . | 0.435 |
| CI | . | -0.009 - 0.045 | . | -0.003 - 0.041 | . | -0.044 - 0.019 |
| **Higher Education** | . | -0.010 | . | -0.003 | . | -0.005 |
| SE | . | 0.014 | . | 0.012 | . | 0.016 |
| T | . | -0.734 | . | -0.258 | . | -0.284 |
| P | . | 0.463 | . | 0.797 | . | 0.776 |
| CI | . | -0.038 - 0.017 | . | -0.026 - 0.020 | . | -0.037 - 0.027 |
| **Working hours mother** | . | 0.002* | . | 0.001* | . | 0.001 |
| SE | . | 0.001 | . | 0.001 | . | 0.001 |
| T | . | 2.269 | . | 2.268 | . | 1.233 |
| P | . | 0.023 | . | 0.023 | . | 0.217 |
| CI | . | 0.000 - 0.003 | . | 0.000 - 0.002 | . | -0.001 - 0.002 |
| **Household income (log)** | . | -0.035 | . | -0.009 | . | -0.038 |
| SE | . | 0.018 | . | 0.016 | . | 0.022 |
| T | . | -1.924 | . | -0.545 | . | -1.737 |
| P | . | 0.054 | . | 0.586 | . | 0.082 |
| CI | . | -0.070 - 0.001 | . | -0.040 - 0.023 | . | -0.080 - 0.005 |
| **Homeowner** | . | -0.015 | . | 0.006 | . | 0.012 |
| SE | . | 0.014 | . | 0.012 | . | 0.017 |
| T | . | -1.067 | . | 0.457 | . | 0.722 |
| P | . | 0.286 | . | 0.648 | . | 0.470 |
| CI | . | -0.043 - 0.013 | . | -0.019 - 0.030 | . | -0.021 - 0.046 |
| **Household size** | . | -0.005 | . | -0.019* | . | -0.001 |
| SE | . | 0.009 | . | 0.008 | . | 0.008 |
| T | . | -0.499 | . | -2.497 | . | -0.081 |
| P | . | 0.618 | . | 0.013 | . | 0.936 |
| CI | . | -0.023 - 0.013 | . | -0.034 - -0.004 | . | -0.016 - 0.015 |
| **Year & Federal State** |  | Yes |  | Yes |  | Yes |
| **R^2^** | 0.029 | 0.111 | 0.028 | 0.200 | <0.001 | 0.068 |
| **N** | 2,141 | 2,128 | 2,141 | 2,141 | 2,141 | 2,141 |
| Standard errors in parentheses. 13 individuals excluded, since one regional fixed effect predicts preterm perfectly. *** p<0.001, ** p<0.01, * p<0.05. Controls are the same variables as in the OLS model (Table 1).  ^a^ Preterm birth.  ^b^ Low birth weight.  ^c^ Small for gestational age. | | | | | | |

*Table S5: OLS Estimates (SOEP)*

| **VARIABLES** | **(1) PT^a^** | **(2) PT^a^** | **(3) LBW^b^** | **(4) LBW^b^** | **(5) SGA^c^** | **(6) SGA^c^** |
| --- | --- | --- | --- | --- | --- | --- |
| **Poor Mental Health** | 0.127*** | 0.095*** | 0.097*** | 0.078*** | 0.019 | 0.007 |
| SE | 0.024 | 0.027 | 0.021 | 0.023 | 0.021 | 0.023 |
| T | 5.265 | 3.531 | 4.519 | 3.414 | 0.909 | 0.290 |
| P | <0.001 | <0.001 | <0.001 | <0.001 | 0.363 | 0.772 |
| CI | 0.080 - 0.175 | 0.042 - 0.147 | 0.055 - 0.139 | 0.033 - 0.123 | -0.022 - 0.061 | -0.039 - 0.053 |
| **Poor Physical Health** | . | 0.046* | . | 0.037* | . | -0.002 |
| SE | . | 0.020 | . | 0.016 | . | 0.019 |
| T | . | 2.322 | . | 2.239 | . | -0.082 |
| P | . | 0.020 | . | 0.025 | . | 0.935 |
| CI | . | 0.007 - 0.085 | . | 0.005 - 0.069 | . | -0.038 - 0.035 |
| **Age Mother** | . | 0.009 | . | 0.005 | . | -0.011 |
| SE | . | 0.012 | . | 0.010 | . | 0.016 |
| T | . | 0.762 | . | 0.475 | . | -0.704 |
| P | . | 0.446 | . | 0.635 | . | 0.482 |
| CI | . | -0.014 - 0.031 | . | -0.015 - 0.025 | . | -0.041 - 0.020 |
| **Age Mother^2^** | . | 0.000 | . | 0.000 | . | 0.000 |
| SE | . | 0.000 | . | 0.000 | . | 0.000 |
| T | . | -0.571 | . | -0.294 | . | 0.903 |
| P | . | 0.568 | . | 0.769 | . | 0.366 |
| CI | . | 0.000 - 0.000 | . | 0.000 - 0.000 | . | 0.000 - 0.001 |
| **Sex** | . | 0.022 | . | 0.000 | . | -0.017 |
| SE | . | 0.013 | . | 0.010 | . | 0.014 |
| T | . | 1.762 | . | -0.019 | . | -1.203 |
| P | . | 0.078 | . | 0.985 | . | 0.229 |
| CI | . | -0.002 - 0.047 | . | -0.021 - 0.020 | . | -0.045 - 0.011 |
| **Twin (multiple birth)** | . | 0.302*** | . | 0.454*** | . | 0.354*** |
| SE | . | 0.061 | . | 0.061 | . | 0.061 |
| T | . | 4.918 | . | 7.434 | . | 5.783 |
| P | . | <0.001 | . | <0.001 | . | <0.001 |
| CI | . | 0.181 - 0.422 | . | 0.334 - 0.574 | . | 0.234 - 0.474 |
| **Previous PT^a^** | . | 0.076 | . | 0.037 | . | 0.065 |
| SE | . | 0.042 | . | 0.028 | . | 0.039 |
| T | . | 1.807 | . | 1.333 | . | 1.668 |
| P | . | 0.071 | . | 0.183 | . | 0.096 |
| CI | . | -0.006 - 0.158 | . | -0.018 - 0.092 | . | -0.011 - 0.142 |
| **Older Sibling** | . | -0.033 | . | -0.027 | . | -0.062*** |
| SE | . | 0.018 | . | 0.014 | . | 0.019 |
| T | . | -1.807 | . | -1.878 | . | -3.346 |
| P | . | 0.071 | . | 0.061 | . | <0.001 |
| CI | . | -0.069 - 0.003 | . | -0.055 - 0.001 | . | -0.098 - -0.026 |
| **Smoked before pregnancy** | . | 0.017 | . | -0.002 | . | 0.021 |
| SE | . | 0.018 | . | 0.014 | . | 0.020 |
| T | . | 0.936 | . | -0.125 | . | 1.064 |
| P | . | 0.349 | . | 0.901 | . | 0.288 |
| CI | . | -0.018 - 0.051 | . | -0.028 - 0.025 | . | -0.018 - 0.059 |
| **Migration background** | . | 0.010 | . | -0.022 | . | -0.023 |
| SE | . | 0.016 | . | 0.013 | . | 0.016 |
| T | . | 0.654 | . | -1.730 | . | -1.435 |
| P | . | 0.513 | . | 0.084 | . | 0.152 |
| CI | . | -0.021 - 0.041 | . | -0.047 - 0.003 | . | -0.055 - 0.009 |
| **Marital Status** | . | 0.016 | . | 0.015 | . | -0.013 |
| SE | . | 0.014 | . | 0.012 | . | 0.016 |
| T | . | 1.140 | . | 1.299 | . | -0.763 |
| P | . | 0.254 | . | 0.194 | . | 0.446 |
| CI | . | -0.012 - 0.044 | . | -0.008 - 0.038 | . | -0.045 - 0.020 |
| **Higher Education** | . | -0.008 | . | 0.001 | . | -0.006 |
| SE | . | 0.014 | . | 0.012 | . | 0.016 |
| T | . | -0.605 | . | 0.056 | . | -0.369 |
| P | . | 0.545 | . | 0.955 | . | 0.712 |
| CI | . | -0.035 - 0.019 | . | -0.022 - 0.024 | . | -0.038 - 0.026 |
| **Working hours mother** | . | 0.001* | . | 0.001* | . | 0.001 |
| SE | . | 0.001 | . | 0.000 | . | 0.001 |
| T | . | 2.389 | . | 2.482 | . | 1.186 |
| P | . | 0.017 | . | 0.013 | . | 0.236 |
| CI | . | 0.000 - 0.003 | . | 0.000 - 0.002 | . | -0.001 - 0.002 |
| **Household income (log)** | . | -0.030 | . | -0.006 | . | -0.038 |
| SE | . | 0.017 | . | 0.015 | . | 0.021 |
| T | . | -1.746 | . | -0.402 | . | -1.793 |
| P | . | 0.081 | . | 0.688 | . | 0.073 |
| CI | . | -0.063 - 0.004 | . | -0.036 - 0.024 | . | -0.080 - 0.004 |
| **Homeowner** | . | -0.015 | . | 0.006 | . | 0.012 |
| SE | . | 0.014 | . | 0.012 | . | 0.016 |
| T | . | -1.108 | . | 0.472 | . | 0.759 |
| P | . | 0.268 | . | 0.637 | . | 0.448 |
| CI | . | -0.043 - 0.012 | . | -0.018 - 0.029 | . | -0.020 - 0.044 |
| **Household size** | . | -0.004 | . | -0.015** | . | -0.000 |
| SE | . | 0.008 | . | 0.005 | . | 0.008 |
| T | . | -0.416 | . | -2.700 | . | -0.019 |
| P | . | 0.677 | . | 0.007 | . | 0.985 |
| CI | . | -0.020 - 0.013 | . | -0.025 - -0.004 | . | -0.016 - 0.016 |
| **Year & Federal State** |  | Yes |  | Yes |  | Yes |
| **R^2^** | 0.022 | 0.086 | 0.018 | 0.154 | <0.001 | 0.060 |
| **N** | 2,141 | 2,141 | 2,141 | 2,141 | 2,141 | 2,141 |

*** p<0.001, ** p<0.01, * p<0.05. Controls are the same variables as in the OLS model (Table 1).

^a^ Preterm birth.

^b^ Low birth weight.

^c^ Small for gestational age.

*Table S6: Mother Fixed Effects Estimates (SOEP)*

| **VARIABLES** | **(1) PT^a^** | **(2) PT^a^** | **(3) LBW^b^** | **(4) LBW^b^** | **(5) SGA^c^** | **(6) SGA^c^** |
| --- | --- | --- | --- | --- | --- | --- |
| **Poor Mental Health** | 0.097*** | 0.062*** | 0.063*** | 0.046* | 0.035 | 0.048 |
| SE | 0.022 | 0.018 | 0.018 | 0.018 | 0.024 | 0.026 |
| T | 4.370 | 3.382 | 3.554 | 2.501 | 1.445 | 1.858 |
| P | <0.001 | <0.001 | <0.001 | 0.013 | 0.149 | 0.063 |
| CI | 0.054 - 0.141 | 0.026 - 0.098 | 0.028 - 0.097 | 0.010 - 0.082 | -0.013 - 0.083 | -0.003 - 0.100 |
| **Poor Physical Health** | . | 0.014 | . | 0.022 | . | 0.001 |
| SE | . | 0.016 | . | 0.016 | . | 0.023 |
| T | . | 0.852 | . | 1.361 | . | 0.038 |
| P | . | 0.394 | . | 0.174 | . | 0.969 |
| CI | . | -0.018 - 0.045 | . | -0.010 - 0.054 | . | -0.044 - 0.046 |
| **Sex** | . | 0.016 | . | -0.001 | . | 0.007 |
| SE | . | 0.011 | . | 0.011 | . | 0.016 |
| T | . | 1.497 | . | -0.116 | . | 0.453 |
| P | . | 0.135 | . | 0.908 | . | 0.651 |
| CI | . | -0.005 - 0.038 | . | -0.023 - 0.020 | . | -0.024 - 0.038 |
| **Previous PT^a^** | . | -0.745*** | . | -0.226*** | . | 0.061 |
| SE | . | 0.026 | . | 0.026 | . | 0.037 |
| T | . | -28.436 | . | -8.562 | . | 1.631 |
| P | . | <0.001 | . | <0.001 | . | 0.103 |
| CI | . | -0.796 - -0.694 | . | -0.278 - -0.174 | . | -0.012 - 0.134 |
| **Older Sibling** | . | 0.045*** | . | 0.002 | . | -0.073*** |
| SE | . | 0.010 | . | 0.010 | . | 0.015 |
| T | . | 4.379 | . | 0.238 | . | -4.917 |
| P | . | <0.001 | . | 0.812 | . | <0.001 |
| CI | . | 0.025 - 0.066 | . | -0.018 - 0.023 | . | -0.102 - -0.044 |
| **R^2^-adjusted** | 0.174 | 0.523 | 0.253 | 0.300 | 0.276 | 0.289 |
| **N** | 2,119 | 2,119 | 2,119 | 2,119 | 2,119 | 2,119 |

*** p<0.001, ** p<0.01, * p<0.05. Controls are the same variables as in the mother fixed effect model (Table 1).^a^ Preterm birth.

^b^ Low birth weight.

^c^ Small for gestational age.

*Table S7: Matching Estimates (SOEP)*

| **VARIABLES** | **(1) PT^a^** | **(2) PT^a^** | **(3) LBW^b^** | **(4) LBW^b^** | **(5) SGA^c^** | **(6) SGA^c^** |
| --- | --- | --- | --- | --- | --- | --- |
| **Poor Mental Health** | ***GAU^f^*** | ***EPA^g^*** | ***GAU^f^*** | ***EPA^g^*** | ***GAU^f^*** | ***EPA^g^*** |
| **ATE^d^** | 0.113* | 0.144*** | 0.130** | 0.091** | 0.031 | -0.009 |
| SE | 0.057 | 0.034 | 0.055 | 0.036 | 0.040 | 0.027 |
| T | 1.990 | 4.230 | 2.380 | 2.520 | 0.780 | -0.610 |
| P | 0.047 | <0.001 | 0.017 | 0.012 | 0.434 | 0.543 |
| CI | 0.002 - 0.224 | 0.077 - 0.210 | 0.023 - 0.236 | 0.020 - 0.162 | -0.047 - 0.109 | -0.070 - 0.037 |
| **ATT^e^** | 0.086** | 0.093** | 0.072** | 0.065** | 0.003 | -0.017 |
| SE | 0.034 | 0.029 | 0.030 | 0.027 | 0.025 | 0.027 |
| T | 2.580 | 3.270 | 2.420 | 2.420 | 0.130 | 1.300 |
| P | 0.010 | 0.001 | 0.015 | 0.016 | 0.895 | 0.193 |
| CI | 0.021 - 0.152 | 0.037 - 0.149 | 0.014 - 0.130 | 0.012 - 0.118 | -0.046 - 0.053 | -0.026 - 0.130 |
| **Support** | 1 | 0.838 | 1 | 0.838 | 1 | 0.838 |
| **N** | 2,134 | 2,134 | 2,134 | 2,134 | 2,134 | 2,134 |

The table presents matching estimates for the average treatment effect (ATE) and average treatment effect on the treated (ATT) of maternal mental health on our birth outcomes (preterm birth, LBW, SGA) using the SOEP sample. We matched all covariates presented in Table S8 as well as survey years and federal states (not indicated in Table S8). The sample size is different, because the mcs scores are added as a covariate to match individuals. *** p<0.001, ** p<0.01, * p<0.05.^a^ Preterm birth.

^b^ Low birth weight.

^c^ Small for gestational age.

^d^ Average Treatment Effect.

^e^ Average Treatment Effect on the Treated.

^f^ Gaussian Kernel.

^g^ Epanechnikov Kernel.

*Table S8: Mean differences before and after matching*

|  | **Unbalanced (before Matching)** | | | **Balanced (after Matching)** | | |
| --- | --- | --- | --- | --- | --- | --- |
| **Variables** | **Poor  mental health** | **Good  mental health** | **p** | **Poor  mental health** | **Good  mental health** | **p** |
| **Poor Physical Health** |  |  |  |  |  |  |
| Gaussian | 0.716 | 0.149 | <0.001 | 0.716 | 0.716 | 0.980 |
| Epanechnikov | 0.716 | 0.149 | <0.001 | 0.708 | 0.708 | 0.992 |
| **Maternal age at birth** |  |  |  |  |  |  |
| Gaussian | 30.829 | 31.434 | 0.055 | 30.829 | 31.197 | 0.404 |
| Epanechnikov | 30.829 | 31.434 | 0.055 | 30.880 | 31.608 | 0.096 |
| **Maternal age at birth^2^** |  |  |  |  |  |  |
| Gaussian | 979.5 | 1012.9 | 0.091 | 979.5 | 1001.9 | 0.412 |
| Epanechnikov | 979.5 | 1012.9 | 0.091 | 982.4 | 1025.5 | 0.113 |
| **Sex** |  |  |  |  |  |  |
| Gaussian | 0.528 | 0.499 | 0.349 | 0.528 | 0.570 | 0.297 |
| Epanechnikov | 0.528 | 0.499 | 0.349 | 0.529 | 0.562 | 0.423 |
| **Twin (multiple birth)** |  |  |  |  |  |  |
| Gaussian | 0.047 | 0.029 | 0.099 | 0.047 | 0.048 | 0.929 |
| Epanechnikov | 0.047 | 0.029 | 0.099 | 0.048 | 0.051 | 0.883 |
| **Previous PT^a^** |  |  |  |  |  |  |
| Gaussian | 0.060 | 0.036 | 0.046 | 0.060 | 0.052 | 0.665 |
| Epanechnikov | 0.060 | 0.036 | 0.046 | 0.052 | 0.052 | 0.965 |
| **Older Sibling** |  |  |  |  |  |  |
| Gaussian | 0.652 | 0.538 | <0.001 | 0.652 | 0.647 | 0.899 |
| Epanechnikov | 0.652 | 0.538 | <0.001 | 0.643 | 0.635 | 0.857 |
| **Smoked before pregnancy** |  |  |  |  |  |  |
| Gaussian | 0.375 | 0.203 | <0.001 | 0.375 | 0.396 | 0.589 |
| Epanechnikov | 0.375 | 0.203 | <0.001 | 0.364 | 0.387 | 0.566 |
| **Migration background** |  |  |  |  |  |  |
| Gaussian | 0.298 | 0.232 | 0.013 | 0.298 | 0.292 | 0.883 |
| Epanechnikov | 0.298 | 0.232 | 0.013 | 0.299 | 0.269 | 0.426 |
| **Marital Status** |  |  |  |  |  |  |
| Gaussian | 0.605 | 0.632 | 0.384 | 0.605 | 0.605 | 0.492 |
| Epanechnikov | 0.605 | 0.632 | 0.384 | 0.608 | 0.622 | 0.737 |
| **Higher Education** |  |  |  |  |  |  |
| Gaussian | 0.355 | 0.518 | <0.001 | 0.355 | 0.372 | 0.661 |
| Epanechnikov | 0.355 | 0.518 | <0.001 | 0.364 | 0.388 | 0.564 |
| **Working hours mother** |  |  |  |  |  |  |
| Gaussian | 32.646 | 34.512 | 0.005 | 32.646 | 31.475 | 0.217 |
| Epanechnikov | 32.646 | 34.512 | 0.005 | 32.708 | 31.874 | 0.380 |
| **Household income (log)** |  |  |  |  |  |  |
| Gaussian | 10.321 | 10.453 | <0.001 | 10.321 | 10.316 | 0.890 |
| Epanechnikov | 10.321 | 10.453 | <0.001 | 10.329 | 10.331 | 0.939 |
| **Homeowner** |  |  |  |  |  |  |
| Gaussian | 0.314 | 0.352 | 0.205 | 0.314 | 0.316 | 0.975 |
| Epanechnikov | 0.314 | 0.352 | 0.205 | 0.313 | 0.339 | 0.505 |
| **Household size** |  |  |  |  |  |  |
| Gaussian | 2.990 | 2.770 | 0.001 | 2.990 | 2.903 | 0.342 |
| Epanechnikov | 2.990 | 2.770 | 0.001 | 2.983 | 2.893 | 0.340 |
| **mcs** |  |  |  |  |  |  |
| Gaussian | 46.274 | 49.383 | <0.001 | 46.274 | 45.584 | 0.396 |
| Epanechnikov | 46.274 | 49.383 | <0.001 | 46.588 | 45.738 | 0.298 |
| T-test for zero mean differences across maternal mental health. The table shows (weighted) means before and those after Gaussian and Epanechnikov matching.  ^a^ Preterm birth. | | | | | | |

*Table S9: OLS Estimates (NEPS)*

| **VARIABLES** | **(1) PT^a^** | **(2) PT^a^** | **(3) LBW^b^** | **(4) LBW^b^** |
| --- | --- | --- | --- | --- |
| **Poor Mental Health** | 0.112*** | 0.093*** | 0.073*** | 0.065** |
| SE | 0.022 | 0.023 | 0.019 | 0.021 |
| T | 5.141 | 4.053 | 3.776 | 3.030 |
| P | <0.001 | <0.001 | <0.001 | 0.002 |
| CI | 0.069 - 0.155 | 0.048 - 0.139 | 0.035 - 0.112 | 0.023 - 0.107 |
| **Poor Physical Health** | . | 0.042** | . | 0.031* |
| SE | . | 0.016 | . | 0.016 |
| T | . | 2.630 | . | 2.007 |
| P | . | 0.009 | . | 0.045 |
| CI | . | 0.011 - 0.074 | . | 0.001 - 0.062 |
| **Age Mother** | . | -0.002 | . | -0.004 |
| SE | . | 0.004 | . | 0.005 |
| T | . | -0.560 | . | -0.865 |
| P | . | 0.576 | . | 0.387 |
| CI | . | -0.011 - 0.006 | . | -0.014 - 0.005 |
| **Age Mother^2^** | . | 0.000 | . | 0.000 |
| SE | . | 0.000 | . | 0.000 |
| T | . | 0.382 | . | 0.731 |
| P | . | 0.702 | . | 0.465 |
| CI | . | 0.000 - 0.000 | . | 0.000 - 0.000 |
| **Sex** | . | 0.013 | . | -0.004 |
| SE | . | 0.011 | . | 0.011 |
| T | . | 1.174 | . | -0.402 |
| P | . | 0.241 | . | 0.688 |
| CI | . | -0.009 - 0.036 | . | -0.026 - 0.017 |
| **Migration background** | . | 0.000 | . | -0.010 |
| SE | . | 0.012 | . | 0.011 |
| T | . | 0.033 | . | -0.921 |
| P | . | 0.974 | . | 0.357 |
| CI | . | -0.023 - 0.024 | . | -0.032 - 0.012 |
| **Higher Education** | . | 0.004 | . | 0.019 |
| SE | . | 0.012 | . | 0.011 |
| T | . | 0.385 | . | 1.687 |
| P | . | 0.700 | . | 0.092 |
| CI | . | -0.018 - 0.027 | . | -0.003 - 0.040 |
| **Working hours mother** | . | -0.000 | . | 0.000 |
| SE | . | 0.001 | . | 0.001 |
| T | . | -0.269 | . | -0.735 |
| P | . | 0.788 | . | 0.463 |
| CI | . | -0.001 - 0.001 | . | -0.002 - 0.001 |
| **Household income (log)** | . | 0.019 | . | 0.037* |
| SE | . | 0.014 | . | 0.015 |
| T | . | 1.300 | . | 2.495 |
| P | . | 0.194 | . | 0.013 |
| CI | . | -0.010 - 0.047 | . | 0.008 - 0.065 |
| **Homeowner** | . | -0.016 | . | -0.019 |
| SE | . | 0.012 | . | 0.012 |
| T | . | -1.318 | . | -1.619 |
| P | . | 0.188 | . | 0.106 |
| CI | . | -0.039 - 0.008 | . | -0.042 - 0.004 |
| **Household size** | . | -0.004 | . | -0.001 |
| SE | . | 0.007 | . | 0.006 |
| T | . | -0.607 | . | -0.131 |
| P | . | 0.544 | . | 0.896 |
| CI | . | -0.017 - 0.009 | . | -0.013 - 0.011 |
| **R^2^** | 0.028 | 0.035 | 0.013 | 0.023 |
| **N** | 1,841 | 1,841 | 1,841 | 1,841 |
| This table presents estimates based on the NEPS data, as described in the article. *** p<0.001, ** p<0.01, * p<0.05.  ^a^ Preterm birth.  ^b^ Low birth weight. | | | | |

*Table S10: DiD with mcs and birth outcomes – SOEP*

| **VARIABLES** | **(1) mcs^d^** | **(2) mcs^d^** | **(3) mcs^d^** | **(4) mcs^d^** | **(5) mcs^d^** | **(6) mcs^d^** |
| --- | --- | --- | --- | --- | --- | --- |
| **PT^a^** | 1.322 | 1.430 | . | . | . | . |
| SE | 0.805 | 0.774 | . | . | . | . |
| T | 1.643 | 1.847 | . | . | . | . |
| P | 0.101 | 0.065 | . | . | . | . |
| CI | -0.256 - 2.900 | -0.088 - 2.948 | . | . | . | . |
| **LBW^b^** | . | . | -0.353 | -0.750 | . | . |
| SE | . | . | 0.975 | 0.982 | . | . |
| T | . | . | -0.362 | -0.764 | . | . |
| P | . | . | 0.717 | 0.445 | . | . |
| CI | . | . | -2.265 - 1.559 | -2.676 - 1.176 | . | . |
| **SGA^c^** | . | . | . | . | -0.986 | -1.445* |
| SE | . | . | . | . | 0.708 | 0.719 |
| T | . | . | . | . | -1.392 | -2.010 |
| P | . | . | . | . | 0.164 | 0.045 |
| CI | . | . | . | . | -2.375 - 0.403 | -2.854 - -0.035 |
| **PT^a^ * After Birth** | -0.187 | -0.446 | . | . | . | . |
| SE | 1.081 | 1.026 | . | . | . | . |
| T | -0.173 | -0.434 | . | . | . | . |
| P | 0.863 | 0.664 | . | . | . | . |
| CI | -2.305 - 1.932 | -2.457 - 1.566 | . | . | . | . |
| **LBW^b^ * After Birth** | . | . | -0.094 | -0.309 | . | . |
| SE | . | . | 1.353 | 1.355 | . | . |
| T | . | . | -0.069 | -0.228 | . | . |
| P | . | . | 0.945 | 0.820 | . | . |
| CI | . | . | -2.747 - 2.559 | -2.965 - 2.348 | . | . |
| **SGA^c^ * After Birth** | . | . | . | . | 1.186 | 1.538 |
| SE | . | . | . | . | 0.971 | 0.969 |
| T | . | . | . | . | 1.222 | 1.588 |
| P | . | . | . | . | 0.222 | 0.112 |
| CI | . | . | . | . | -0.718 - 3.089 | -0.361 - 3.438 |
| **After Birth** | 0.926** | -0.229 | 0.898** | -0.270 | 0.736* | -0.458 |
| SE | 0.354 | 0.605 | 0.346 | 0.603 | 0.360 | 0.609 |
| T | 2.619 | -0.378 | 2.593 | -0.448 | 2.046 | -0.752 |
| P | 0.009 | 0.705 | 0.010 | 0.655 | 0.041 | 0.452 |
| CI | 0.233 - 1.620 | -1.415 - 0.957 | 0.219 - 1.577 | -1.452 - 0.912 | 0.031 - 1.441 | -1.653 - 0.736 |
| **pcs^e^** | -0.141*** | -0.133*** | -0.143*** | -0.137*** | -0.143*** | -0.135*** |
| SE | 0.027 | 0.027 | 0.027 | 0.027 | 0.027 | 0.027 |
| T | -5.190 | -4.871 | -5.259 | -4.974 | -5.265 | -4.954 |
| P | <0.001 | <0.001 | <0.001 | <0.001 | <0.001 | <0.001 |
| CI | -0.194 - -0.088 | -0.187 - -0.080 | -0.197 - -0.090 | -0.191 - -0.083 | -0.196 - -0.090 | -0.189 - -0.082 |
| **Age Mother** | . | -1.024*** | . | -1.022*** | . | -1.036*** |
| SE | . | 0.227 | . | 0.227 | . | 0.227 |
| T | . | -4.509 | . | -4.504 | . | -4.566 |
| P | . | <0.001 | . | <0.001 | . | <0.001 |
| CI | . | -1.469 - -0.579 | . | -1.466 - -0.577 | . | -1.481 - -0.591 |
| **Age Mother^2^** | . | 0.015*** | . | 0.015*** | . | 0.015*** |
| SE | . | 0.003 | . | 0.003 | . | 0.003 |
| T | . | 4.732 | . | 4.733 | . | 4.797 |
| P | . | <0.001 | . | <0.001 | . | <0.001 |
| CI | . | 0.009 - 0.021 | . | 0.009 - 0.021 | . | 0.009 - 0.022 |
| **Sex** | . | -0.950** | . | -0.938** | . | -0.950** |
| SE | . | 0.328 | . | 0.328 | . | 0.328 |
| T | . | -2.901 | . | -2.856 | . | -2.894 |
| P | . | 0.004 | . | 0.004 | . | 0.004 |
| CI | . | -1.593 - -0.308 | . | -1.582 - -0.294 | . | -1.594 - -0.306 |
| **Twin (multiple birth)** | . | 0.184 | . | 0.989 | . | 0.724 |
| SE | . | 1.115 | . | 1.149 | . | 1.115 |
| T | . | 0.165 | . | 0.861 | . | 0.649 |
| P | . | 0.869 | . | 0.389 | . | 0.516 |
| CI | . | -2.002 - 2.370 | . | -1.264 - 3.243 | . | -1.463 - 2.911 |
| **Previous PT^a^** | . | 2.565*** | . | 2.690*** | . | 2.687*** |
| SE | . | 0.731 | . | 0.728 | . | 0.727 |
| T | . | 3.510 | . | 3.693 | . | 3.695 |
| P | . | <0.001 | . | <0.001 | . | <0.001 |
| CI | . | 1.132 - 3.998 | . | 1.262 - 4.118 | . | 1.261 - 4.113 |
| **Older Sibling** | . | -0.948* | . | -1.045** | . | -1.051** |
| SE | . | 0.403 | . | 0.403 | . | 0.404 |
| T | . | -2.352 | . | -2.590 | . | -2.604 |
| P | . | 0.019 | . | 0.010 | . | 0.009 |
| CI | . | -1.739 - -0.158 | . | -1.836 - -0.254 | . | -1.843 - -0.260 |
| **Smoked before pregnancy** | . | -0.551 | . | -0.519 | . | -0.511 |
| SE | . | 0.395 | . | 0.396 | . | 0.396 |
| T | . | -1.392 | . | -1.312 | . | -1.291 |
| P | . | 0.164 | . | 0.190 | . | 0.197 |
| CI | . | -1.326 - 0.225 | . | -1.295 - 0.257 | . | -1.287 - 0.265 |
| **Migration background** | . | 0.958 | . | 0.946 | . | 0.940 |
| SE | . | 0.497 | . | 0.500 | . | 0.499 |
| T | . | 1.929 | . | 1.892 | . | 1.883 |
| P | . | 0.054 | . | 0.059 | . | 0.060 |
| CI | . | -0.016 - 1.932 | . | -0.034 - 1.926 | . | -0.039 - 1.919 |
| **Marital Status** | . | 1.402*** | . | 1.418*** | . | 1.408*** |
| SE | . | 0.415 | . | 0.418 | . | 0.416 |
| T | . | 3.374 | . | 3.395 | . | 3.386 |
| P | . | 0.001 | . | 0.001 | . | 0.001 |
| CI | . | 0.587 - 2.216 | . | 0.599 - 2.237 | . | 0.593 - 2.224 |
| **Higher Education** | . | -0.068 | . | -0.144 | . | -0.114 |
| SE | . | 0.360 | . | 0.359 | . | 0.359 |
| T | . | -0.188 | . | -0.402 | . | -0.316 |
| P | . | 0.851 | . | 0.688 | . | 0.752 |
| CI | . | -0.774 - 0.639 | . | -0.849 - 0.560 | . | -0.818 - 0.590 |
| **Working hours mother** | . | -0.031* | . | -0.030* | . | -0.029 |
| SE | . | 0.015 | . | 0.015 | . | 0.015 |
| T | . | -2.045 | . | -1.981 | . | -1.910 |
| P | . | 0.041 | . | 0.048 | . | 0.056 |
| CI | . | -0.062 - -0.001 | . | -0.061 - 0.000 | . | -0.060 - 0.001 |
| **Household income (log)** | . | 2.041*** | . | 2.081*** | . | 2.058*** |
| SE | . | 0.429 | . | 0.429 | . | 0.429 |
| T | . | 4.759 | . | 4.855 | . | 4.802 |
| P | . | <0.001 | . | <0.001 | . | <0.001 |
| CI | . | 1.200 - 2.882 | . | 1.241 - 2.921 | . | 1.218 - 2.898 |
| **Homeowner** | . | -0.544 | . | -0.583 | . | -0.557 |
| SE | . | 0.373 | . | 0.373 | . | 0.373 |
| T | . | -1.457 | . | -1.561 | . | -1.494 |
| P | . | 0.145 | . | 0.119 | . | 0.135 |
| CI | . | -1.277 - 0.188 | . | -1.315 - 0.149 | . | -1.288 - 0.174 |
| **Household size** | . | -0.300 | . | -0.326 | . | -0.315 |
| SE | . | 0.238 | . | 0.238 | . | 0.237 |
| T | . | -1.262 | . | -1.366 | . | -1.327 |
| P | . | 0.207 | . | 0.172 | . | 0.185 |
| CI | . | -0.766 - 0.166 | . | -0.793 - 0.142 | . | -0.780 - 0.150 |
| **Year & Federal State** |  | Yes |  | Yes |  | Yes |
| **R^2^** | 0.017 | 0.073 | 0.015 | 0.072 | 0.016 | 0.073 |
| **N** | 3,123 | 3,123 | 3,123 | 3,123 | 3,123 | 3,123 |

The table shows DiD estimates for the relationship between preterm birth and the mcs score of the mother after birth using the SOEP sample. We only included births with at least one nonmissing maternal mcs score before and after birth. All models also include the physical health component score (pcs) as a control variable (not indicated below). Robust standard errors in parenthesis. *** p<0.001, ** p<0.01, * p<0.05.

^a^ Preterm birth.

^b^ Low birth weight.

^c^ Small for gestational age.

^d^ Mental health component score

^e^ Physical health component score.

*Table S11: Samples, controls, outcomes and methods for the specification curve analysis*

| **Controls** | |
| --- | --- |
| First_Control | - Maternal smoking - Region and year fixed effects |
| Second_Control | - Sex (child) - Maternal age at birth (and squared) |
| Third_Control | - Average maternal working hours before birth - Logarithmic average household income before birth - Higher education (mother) |
| Fourth_Control | - Marital status (mother) - Indirect migration background - Homeownership - Number household members |
| Fifth_Control | - Older sibling - Preterm Sibling - Twin/multiple birth |
| **Outcomes** | |
| Birth outcomes | - Preterm birth (preterm) - Low birth weight (lbw) - Low birth weight for gestational age (German standard) (sga_ger) - Small for gestational age (German standard) (sga_height_ger) - Low birth weight for gestational age (intergrowth standard) (sga_inter) - Small for gestational age (intergrowth standard) (sga_height_inter) |
| **Sample** | |
| General samples | - Sample excluding all missing values (OLS_sample) - Sample excluding all missing values expect of maternal smoking (no_smoking) - Sample excluding all missing values expect of maternal working hours (no_working_hours) - Sample excluding all twin observations (no_twins) |
| MOFE sample | - Sample excluding all children without siblings and multiple births used for mother fixed effects models. (MOFE_sample) |
| **Identification** | |
| OLS | - Linear regression model |
| MOFE | - Mother fixed effect model |
| MATCHING | - Gaussian Matching (GAU) - Epanechnikov Matching (EPA) - Both matched with mcs-score (mcs) before birth and without |


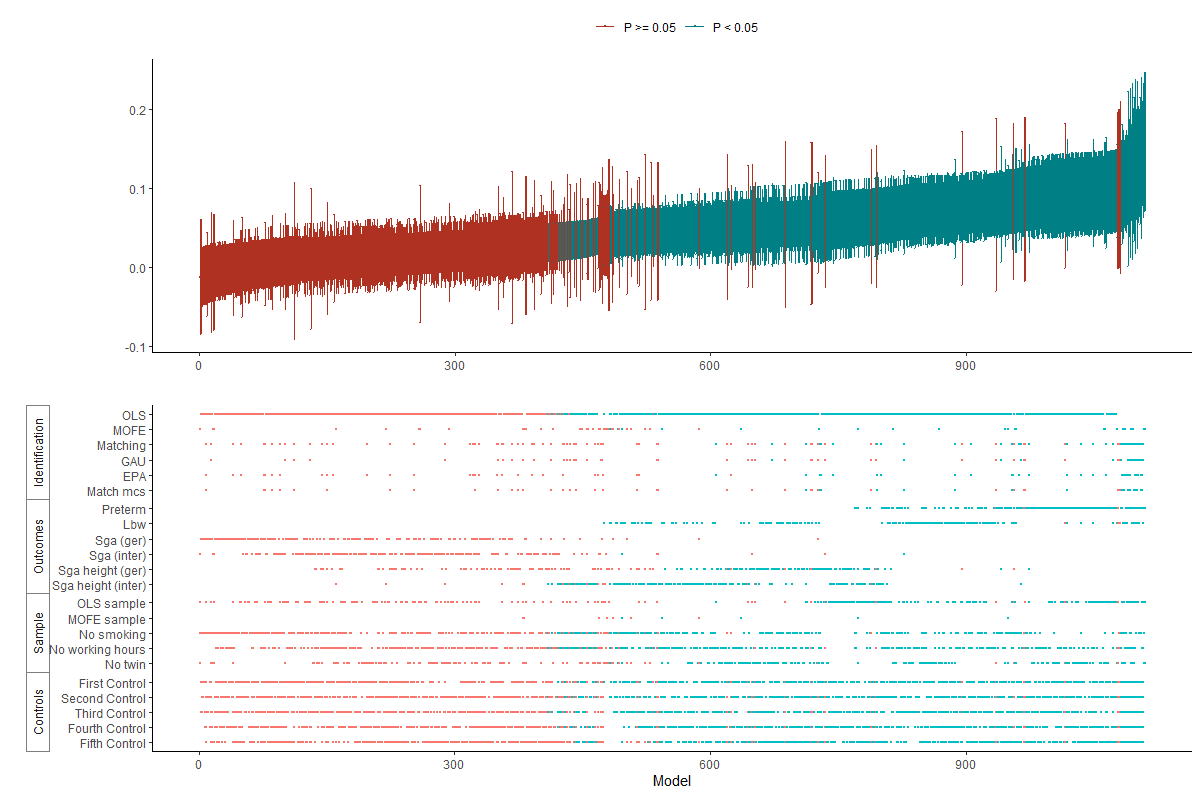


The upper panel shows point estimates and their respective 95% confidence intervals for the different models described below the table. Estimates are ordered from the smallest to the largest. Confidence intervals are calculated using heteroscedasticity robust standard errors.

*Figure S2: Specification curve*
